# Dysphagia education in Addis Ababa, Ethiopia: student self-competency ratings during their dysphagia course

**DOI:** 10.1186/s12909-025-07365-7

**Published:** 2025-05-21

**Authors:** Sana Smaoui, Sandhya Ganesan, Trish Williams

**Affiliations:** 1 Department of Hearing and Speech Sciences, Faculty of Allied Health Sciences, Kuwait University, Kuwait City, Kuwait; 2 Li Ka Shing Knowledge Institute, St. Michael’s Hospital, Unity Health Toronto, Toronto, ON Canada; 3 Department of Speech-Language Pathology, Faculty of Medicine, University of Toronto, Toronto, ON Canada; 4 Division of Otolaryngology-Head and Neck Surgery, The George Washington University School of Medicine & Health Sciences, Washington, DC USA; 5 The Renaissance School of Medicine at Stony Brook University, Stony Brook, NY USA; 6 Toronto General Hospital, University Health Network, Toronto, ON Canada

**Keywords:** Dysphagia, Ethiopia, Speech-language therapy

## Abstract

**Purpose:**

Training programs focused on dysphagia have been identified as an area needing improvement due to the specialized skills required to provide clinical care to patients with dysphagia. Globally, a lack of standardized training has been recognized and has led to the introduction of competency and training frameworks in the clinical practice of dysphagia. Previous studies have explored the experiences of students in training programs and their self-perceived competency; however, none have explored this in the Ethiopian context due to the infancy of the Speech Language Therapy (SLT) profession within this region. The objectives of this study were to explore patterns in self-perceived competency ratings for SLT students at AAU and determine the impact of clinical experiences during student training.

**Methods:**

First- and second-year students enrolled in the two-year SLT Master's program in Ethiopia were taught the dysphagia course in a combined class in English by visiting faculty. A modified Dysphagia Competency Verification Tool (DCVT) was used to assess self-perception of competency in dysphagia. The tool was administered in April 2024, before any dysphagia-related clinical exposure occurred and once again in May 2024, after clinical exposure to patients with dysphagia occurred. Generalized estimating equations (GEE) models were used for the General Skills (DCVT-GS) and Direct Patient Care (DCVT-DPC) subtests to study variations in responses for self-perceived competency. The models included covariates of sex, background in SLT, dysphagia-specific patient exposure and a repeated factor of survey timepoint.

**Results:**

In total, 38 responses were collected across two time points and from all 19 participants. The SLT students were mostly female (*n* = 16; 84%) with ages ranging from 21 to 46 years. The GEE model for DCVT-GS identified significant main effects of background in SLT (*p* = 0.018), dysphagia patient exposure (*p* = 0.019), and survey timepoint (*p* < 0.001). The GEE model for DCVT-DPC demonstrated significance for background in SLT (*p* < 0.001), dysphagia patient exposure (p = 0.009), and sex (*p* = 0.031).

**Conclusion:**

Regardless of DCVT domain, SLT graduate student clinicians were more likely to perceive themselves as “adequate” in their ratings at the second timepoint following clinical interactions, if they had prior SLT experience, including prior dysphagia experience. Training programs exploring dysphagia competency are encouraged to provide increased exposure to patients with dysphagia to support increased self-perceived competency scores.

**Supplementary Information:**

The online version contains supplementary material available at 10.1186/s12909-025-07365-7.

## Introduction

Dysphagia refers to dysfunction in the sensory and/or motor mechanisms that impact the safety and efficiency of swallowing at any point during its stages. Dysphagia can also negatively impact patient quality of life and lead to a slew of negative health outcomes including malnutrition, dehydration, and even death [8]. Globally, the prevalence of dysphagia ranges from 2 to 20% [9]. In North America, certified Speech Language Therapists (SLTs) are the preferred providers for direct clinical management and treatment for patients with dysphagia. Despite the initial establishment of an undergraduate Speech and Language Therapy (SLT) program at Addis Ababa University (AAU) in Ethiopia, Speech and Language Therapists (SLTs) do not practice in the area of dysphagia at the Black Lion Hospital, Ethiopia's largest, tertiary-level hospital, located in Addis Ababa, Ethiopia.

Currently, there is a gap in the need for speech-language therapy services and access to trained SLT providers in Ethiopia. Specifically, within the adult population in Ethiopia, stroke is one of the largest contributors to long-term morbidity and mortality, with reports of up to 43% of stroke patients at an Ethiopian institution experiencing dysphagia [10–12]. This, coupled with Ethiopia’s aging population, results in a significant need for the evaluation and treatment of dysphagia. The establishment of the SLT Master’s program at AAU is aimed at bridging this gap and providing specialized care for patients with speech, language, and swallowing disorders; however, trained SLTs in Addis Ababa do not currently routinely practice in the area of dysphagia. As a result, there is a need for trained dysphagia specialists from outside of Ethiopia to lead the instruction of dysphagia-related care for students in the SLT Master’s program at AAU.

This underserved SLT service impacts both patients with dysphagia visiting the Black Lion Hospital who are unable to receive SLT services, as well as the competency of SLTs who lack an environment to train and provide mentored dysphagia management and treatment. The evolving nature of the profession necessitated the creation of an SLT graduate program to further prepare students to gain clinical and preclinical experience. However, currently, the gap in availability of clinical instructors who would be able to teach graduate-level courses and address the clinical questions that students would need specialized training in. To address this gap, Addis Ababa University (AAU), in partnership with the Black Lion Hospital, the Toronto Addis Ababa Academic Collaboration, and Transforming Faces, established the first master’s level SLT program in which students received training from visiting faculty who were content experts providing targeted teaching in each area in the SLT profession. This program included a dysphagia course where first-year and second-year students enrolled in the two-year SLT Master's program at AAU were taught the dysphagia course in a combined class.

Globally, training programs focused on dysphagia have been identified as an area needing improvement due to the specialized skills necessary to competently provide direct clinical care to patients with dysphagia. A lack of standardization in training across the world has been identified as a theme and has consequently led to the introduction of competency and training frameworks in the clinical practice of dysphagia [1]. Graduate programs in speech-language therapy are not required to provide clinical experiences in the area of dysphagia evaluation and treatment. A 2016 survey conducted by the American Speech and Hearing Association (ASHA) of certified Speech Language Pathologists in the United States who specialize in dysphagia revealed that 37.2% reported having “less than a semester of their graduate training spent on the evaluation and treatment of dysphagia” and 19.8% reported having “no experience with dysphagia management” during the duration of their clinical practicums [2]. Minimum standards have been established to tackle this lack of standardization, including the development of metrics to measure clinical competency in dysphagia management.

A subcommittee of ASHA composed of members from the ASHA Special Interest Group for Swallowing and Swallowing Disorders (SIG 13) and the American Board of Swallowing and Swallowing Disorders (ABSSD) developed the Dysphagia Competency Verification Tool (DCVT). This tool standardizes the evaluation and documentation of clinical competency in dysphagia for certified SLTs. The tool is a metric that incorporates domains of dysphagia management that can be examined to ensure clinical competency in dysphagia management for practicing SLTs [3].

The original DCVT has been explored as a tool for graduate student clinicians to self-assess their skills during their education and clinical experiences [2]. Use of this tool can aid instructors in understanding the alignment of student self-perceptions of competency and actual competency of knowledge and skills. Clinical competency encompasses more than direct clinical skills and task completion. Self-perception of clinical competence encompasses multiple factors including: confidence, self-efficacy, exposure to clinical skills, anxiety levels, and the support provided by graduate instructors [2]. Holistic research on self-perceived competency in clinical professions that considers these factors can provide well-rounded insight into students’ self-awareness of their skillset and encourage self-reflection about areas of growth [4–6]. This information is essential to inform the development of appropriate training throughout the graduate curriculum. Previous studies have explored the experiences of students in dysphagia training programs and the importance of self-perceived competency; however, none have explored this in the Ethiopian context due to the infancy of the SLT profession within this region [7].

The objectives of this study were:

1. To explore patterns in self-perceived competency ratings for SLT students at AAU and determine the impact of clinical experiences during student training.
2. To understand the influence of primary independent variables (sex, background in SLT, previous dysphagia patient exposure) on these ratings.

It is hypothesized that SLT students with a background in SLT and previous dysphagia patient exposure will have increased self-perceived competency ratings on the DCVT.

## Methods

Ethics/Institutional Review Board Committees was obtained from both Addis Ababa University (Ref.No.NEURO/051/16) and The George Washington University (NCR245792). Visiting faculty from the University of Toronto and The George Washington University provided joint theoretical classroom instruction and small group in-person clinical teaching during an intensive (8 h per day) period from April 2024 to May 2024 at the Black Lion Hospital. The course was taught in English; however, English is not the primary language of the students who had a variety of language backgrounds (e.g. Amharic, Afar, Oromo, etc.). The course was divided into two halves, with the first half focused on theoretical classroom instruction and the second half focused on application of dysphagia knowledge in a clinical setting. The topic areas covered in the course are summarized in Table 1.

**Table 1:** Structure of the one-month dysphagia course

First Half – Classroom Instruction (*All day*)	Second Half – Classroom and Clinical Instruction (*modelling, co-assessment, co-treatment, and management of the patients*)
• Theoretical dysphagia coursework topics included: ◦ Anatomy, Physiology, and Neuroanatomy Review ◦ Abnormal Swallow ◦ Dysphagia Screening ◦ Assessment in Dysphagia ◦ Texture modification/IDDSI ◦ Clinical Bedside Swallow Assessment ◦ Documentation & Report Writing ◦ Sharing Assessment Results ◦ Dysphagia Intervention ◦ Pediatric Dysphagia ◦ Esophageal Dysphagia ◦ Instrumental Assessment (VFSS and FEES) ◦ Patient Reported Outcome Measures ◦ Critical Appraisal of Dysphagia ◦ Literature (FRONTIERS) ◦ End of Life – Counseling	• Continued morning classroom instruction • Assessment of neurology inpatients and outpatients with dysphagia symptoms (e.g. stroke, Alzheimer's disease, myasthenia gravis, and Parkinson’s disease) including the following hands-on training: ◦ comprehensive clinical history taking ◦ oral motor examination ◦ clinical bedside swallowing assessments ◦ recommendations for individualized dysphagia management and intervention ▪ diet texture modifications ▪ postural adjustments ▪ swallowing exercises ◦ patient and family education ◦ debrief discussion of clinical takeaways (up to four 4 hours)

### Dysphagia competency verification tool scoring

SLT graduate students rated their self-perceived competency based on the DCVT Clinical Swallow Assessment & Dysphagia Treatment Section. This section includes the key competencies for evaluation and treatment of dysphagia. The specific domains explored were the General Skills (DCVT-GS) and Direct Patient Care (DCVT-DPC) sections. The DCVT-GS focused on general items, such as describing the etiology contributing to feeding and/or swallowing disorders. The DCVT-DPC, however, focused on identifying when swallowing assessment and intervention is appropriate along with population and setting-specific skills, for instance, describing best practices for intervention given complications or medical conditions which may impact individuals'feeding and swallowing.

Previous research has explored differences in the DCVT self-perceived competence in the original binary form (e.g. competent vs inadequate), and using a modified DCVT (absent, dependent, emerging, adequate, excellent). Further modification to the DCVT was made by our team to prevent potential errors due to language barriers (See supplementary file for the modified DCVT questionnaire). Therefore, the modified DCVT ratings in this study were made using a 5-point ordinal scale to better capture changes in self-perceived competency using the following categories: [1] strongly disagree, [2] disagree, neutral, [3] agree, and [4] strongly agree. The following variables were collected in addition to the modified DCVT scores: students’ age, sex, cohort year, length of time as an SLT/SLT student, and number of experiences in assessment, management, and treatment of patients with dysphagia.

### Statistical analyses and data handling

Each individual response on the modified DCVT was reclassified as either Emergent (scores < 4; Strongly Disagree, Disagree, Neutral) or Adequate (scores > 4; Agree, Strongly Agree) as can be seen in Table 2. This grouping allowed for aggregating responses and binary statistical analyses to explore patterns in self-perceived competency. Generalized estimating equations (GEE) models were used for each domain (DCVT-GS and DCVT-DPC) to study variations in responses for self-perceived competency. Primary variables include the modified DCVT responses, sex, background in SLT, previous dysphagia patient exposure, and a repeated factor of survey timepoint. Based on previous literature with student learners [2], we suspect that the SLT students would continue to perceive their competence as inadequate and that, with increases in clinical exposure, we would see changes in perceived competency.

**Table 2:** Modified DCVT Scale with corresponding grouping

**Modified DCVT Rating Scale**	**Description**	**Statistical Handling **
1	Strongly Disagree	Emergent
2	Disagree	
3	Neutral	
4	Agree	Adequate
5	Strongly Agree	

The tools were administered 1) in April 2024, *before* any dysphagia-related real-life clinical exposure was provided during the course and once again 2) in May 2024, *after* real-life clinical encounters with patients being assessed for dysphagia to the Neurology unit at the Black Lion Hospital. Responses were aggregated to explore patterns in self-perceived competency ratings and determine the impact of patient clinical experiences for students with limited exposure to patients with dysphagia compared to those who may have had more experience and exposure to these patients.

## Results

This primary prospective study included 19 first and second-year students enrolled in the first graduate (masters-level) SLT program at Addis Ababa University. In total, 38 responses were collected across two timepoints. As can be seen in Table 3, the SLT students were mostly female (*n* = 16; 84%) with ages ranging from 21 to 46 years. The distribution of undergraduate educational backgrounds was as follows: 37% had obtained speech language therapy undergraduate degrees (*n* = 7), 26% majored in nursing (*n* = 5), 16% majored in special needs education (*n* = 3), and 20% had other degrees (including public health, doctor of medicine, physiotherapy, and English language).

As some students had undergone SLT undergraduate training programs, we were interested in their previous dysphagia patient experience. Responses to this question indicated that 63% (*n* = 12) responded with having had no dysphagia related patient experience prior to the dysphagia course, 15% with less than three dysphagia related patient experiences (*n* = 3), and 21% with 15 or more patient experiences (*n* = 4).

**Table 3:** Demographic information

	**Total** **N (%)**
**Age**	
mean	30 years
range	21—46 years
**Sex**	
Female	16 (84)
Male	3 (16)
**Undergraduate Training**	
Speech-Language Therapy	7 (37)
Nursing	5 (26)
Public Health	1 (5)
Special Needs Education	3 (16)
English Language	1 (5)
Physiotherapy	1 (5)
Doctor of Medicine	1 (5)
**Previous Dysphagia Patient Exposure**	
Yes	7 (37)
No	12 (63)
**Graduate Program Year of Study**	
Year 1	3 (16)
Year 2	16 (84)

Responses for each question posed in the DCVT are noted in Table 4. For the DCVT-GS, the areas that most students marked as Emergent at time point 1 were related to describing research on normal swallowing (Q1), describing the effect of medications on swallowing (Q5) and describing candidacy for instrumental assessment (Q14). Following the clinical interactions, at time point 2, many of these shifted to at least half for each question demonstrating lower overall emergent rankings. For the DCVT-DPC, the areas with most Emergent responses were prognostication (Q16), tailoring plans to the educational level of patients (Q24), staff education (Q18) and discharge criteria (Q25). Similarly to the DCVT-GS, the Emergent responses in time point 1 decreased to less than half at time point 2.

**Table 4:** Dysphagia Competency Verification Tool Rankings per Question

		**Emergent Rankings**			**Adequate Rankings**	
		**T1**	**T2**		**T1**	**T2**
**Dysphagia Competency Verification Tool - General Skills (DCVT – GS)**						
Q1	Describes relevant research on normal swallowing	13	5		6	14
Q2	Explains strengths and limitations of clinical examination, including ability to detect aspiration and determine treatment strategies for pharyngeal swallowing disorders	6	0		13	19
Q3	Describes the etiology contributing to feeding and/or swallowing disorders	2	0		17	19
Q4	Identifies cognitive, communication, behavioral, and psychological factors contributing to feeding and/or swallowing status	4	1		15	18
Q5	Describes the potential effects of common medications on swallowing	16	7		3	12
Q6	Describes the interrelationships of the oral, pharyngeal, and esophageal phases of swallowing	1	0		18	19
Q7	Describes cross-system relationships that influence feeding and/or swallowing (e.g., respiratory, gastrointestinal, neurological)	1	0		18	19
Q8	Identifies signs and symptoms of feeding and/or swallowing disorders	1	0		18	19
Q9	Describes nutritional intake methods (oral and non-oral) and the problems associated with each that may contribute to dysphagia or be exacerbated by dysphagia	5	1		14	18
Q10	Collaborates with relevant team members regarding patient care	4	1		15	18
Q11	Describes and integrates evidence-based practice into patient assessment and care	8	0		11	19
Q12	Recognizes medical contraindications of proceeding with direct assessment, signs of patient distress, and necessary response	7	4		12	15
Q13	Describes differences between screening and assessment	4	0		15	19
Q14	Describes indications and contraindications for instrumental swallow study referral	14	0		5	19
**Dysphagia Competency Verification Tool - Direct Patient Care (DCVT – DPC)**						
Q1	Obtains comprehensive medical and dysphagia history, including nature and duration of signs and symptoms, prior dysphagia evaluation or treatment, response to treatment, and cultural and/or linguistic factors that may influence the patient’s preferences and attitudes toward feeding and/or swallowing	1	0		18	19
Q2	Determines baseline and current nutritional intake (e.g., positioning, feeding dependency, environment, diet modification, compensations)	3	0		16	19
Q3	Identifies when swallowing assessment and intervention is appropriate	8	0		11	19
Q4	Conducts an oral, pharyngeal, laryngeal, cranial nerve, and respiratory function examination as it relates to functional assessment of feeding and/or swallowing	1	0		18	19
Q5	Identifies abnormal/atypical structure and function	4	1		15	18
Q6	Assembles the appropriate assessment materials (e.g., nipples, bottles, utensils, cups, foods/liquids) as per facility-specific protocol	8	1		11	18
Q7	Identifies significant signs, symptoms, medical conditions, and medications pertinent to dysphagia during clinical assessment	3	0		16	19
Q8	Recognizes clinical signs and symptoms of airway compromise	4	2		15	17
Q9	Tests interventions, including but not limited to postural changes, behavioral changes, maneuvers, bolus modifications (e.g., texture, volume), delivery method (e.g., spoon, cup, bottle, nipple type), and sensory enhancement techniques to improve safety and efficiency of the swallow and trials, as appropriate	6	1		13	18
Q10	Refers for appropriate diagnostic tests, including instrumental swallow assessment, and consultations when indicated	10	2		9	17
Q11	Provides recommendations regarding delivery of nutrition and hydration (oral, non-oral, or combination of the two)	9	3		10	16
Q12	Provides recommendations regarding specific oral intake modifications (e.g., volume, viscosity, texture, etc.)	5	0		14	19
Q13	Provides recommendations regarding compensatory and feeding precautions (e.g., strategies, positioning, assistance, supervision, etc.)	7	0		12	19
Q14	Provides recommendations regarding rehabilitation treatment targeting physiologic deficits identified on assessment, utilizing evidence-based techniques when available	8	0		11	19
Q15	Integrates and adapts plan of care to include patient’s cultural and personal preferences	4	0		15	19
Q16	Provides a prognostic statement	14	3		5	16
Q17	Educates the patient and family/caregiver to the findings and recommendations, including options and relative risks/benefits	7	0		12	19
Q18	Educates the staff (e.g., physicians, nurses/CNAs, care planning team, teachers, aides) as to findings and recommendations, and advocates for swallowing-related services	11	1		8	18
Q19	Generates documentation that is clear, concise, complete, and interpretive (e.g., assessment performed/findings, impression, severity, prognosis, recommendations, and goals)	10	1		9	18
Q20	Identifies necessary follow-up care, including frequency of treatment, monitoring, and/or reevaluation	7	2		12	17
Q21	Provides ongoing assessment and revises treatment goals as appropriate, based on patient response	8	0		11	19
Q22	Develops and implements treatment plan targeting physiologic deficits identified on assessment	10	0		9	19
Q23	Documents response to treatment using objective and measurable data collection systems	10	0		9	19
Q24	Adjusts treatment plan, content and delivery to the level of the person being educated, counseled, or trained	12	0		7	19
Q25	Identifies discharge/dismissal criteria	11	4		8	15
Q26	Seeks assistance and collaboration as needed in the assessment and care of persons with dysphagia	5	1		14	18
Q27	Describes best practices for providing interventions when complicated and/or special medical conditions are seen which may have an impact on an individual’s feeding and swallowing	10	3		9	16

*T1 *Timepoint 1, *T2 *Timepoint 2

Given the inherent differences in the duration that each of the cohorts spent in the program which may have impacted their response patterns, we further categorized the results of the first-year students compared to the second-year students per study timepoint in Table 5 to explore a comparison between each cohort. The mean scores for each component of the DCVT appeared comparable across timepoint and DCVT component for both types of scores (emergent and adequate). Interestingly, the second-year students rated both DCVT components with higher means for emergent scores at timepoint 1 compared to the first-year students, however following patient exposure the second-year students ultimately reported higher means for adequate ratings compared to first-year students.

To determine the significance of each variable on responses as a whole, two GEE models were developed, one each for DCVT-GS and DCVT-DPC. The GEE model for DCVT-GS identified significant main effects of background in SLT (*p* = 0.018), dysphagia patient exposure (*p *= 0.019), and survey timepoint (*p* < 0.001). In terms of DCVT-DPC, the second GEE model demonstrated significance for background in SLT (*p* < 0.001), dysphagia patient exposure (*p* = 0.009), and sex (*p* = 0.031).

**Table 5:** Comparison of First and Second Year SLT Students

	First Year SLT Students		Second Year SLT Students	
	Timepoint 1 (T1)Mean (%)	Timepoint 2 (T2)Mean (%)	Timepoint 1 (T1)Mean (%)	Timepoint 2 (T2)Mean (%)
***DCVT – GS (/14)***				
Emergent Scores	4.33 (31%)	1.17 (8%)	4.81 (34%)	0.875 (6%)
Adequate Scores	9.67 (69%)	12.83 (92%)	9.19 (66%)	13.13 (94%)
***DCVT – DPC (/27)***				
Emergent Scores	8.67 (32%)	1.67 (6%)	10.63 (39%)	1.25 (5%)
Adequate Scores	18.33 (68%)	25.33 (94%)	16.38 (61%)	25.75 (95%)
*DCVT-GS* Dysphagia Competency Verification Tool-General Skills, *DCVT- DPC *Dysphagia Competency Verification Tool-Direct Patient Care, *Emergent Scores *1–3 *Adequate Scores* 4–5				

## Discussion

We demonstrate the adaptation of tools developed by ASHA for the evaluation of—and second-year learners in the SLT Master’s program at AAU. The results of the present study are consistent with the hypothesis that a background in SLT and previous dysphagia patient exposure correlate with increased self-perceived competency ratings on the DCVT. Previous studies have demonstrated the value of self-competency tools for both learners and instructors in identifying areas of dysphagia management practice and skills that require further development. Hazelwood et. al. modified the DCVT rating scale to reflect increased variation in self-perceived competence not captured in the binary rating options of “competent” or “inadequate” as in the original scale [2]. We further modified the scale, which included responses of “absent,” “dependent,” “emerging,” “adequate,” and “excellent,” to a 5-point agree/disagree Likert scale. This not only allowed us to capture variation in self-perceived competence but also to account for an English language barrier. Although the use of English instruction is standard at AAU, most students are second-language English speakers, with Amharic and other local languages as their first language. The adjustment of rating terms from a 6 th-8 th grade reading level to a 4 th-6 th grade reading level allows for easier interpretation of these terms and subsequently a more accurate self-assessment of competency.

The modified DCVT survey indicated that an SLT background and dysphagia patient exposure were significantly associated with greater self-assessment scores related to general skills and direct patient care [13]. Students with an SLT background, specifically those who had completed the undergraduate SLT program at Addis Ababa University, were likely exposed to dysphagia-related content in their undergraduate studies. Pre-clinical undergraduate dysphagia education, including courses such as “​​Anatomy for Human Communication and Swallowing” and “Adult Motor Speech and Swallowing,” provide an opportunity for these students to build a foundation of normal swallowing mechanics and become exposed to the area of swallowing disorders [7]. This theoretical foundation may allow them to better integrate clinical dysphagia content into their knowledge base and apply this knowledge in direct patient care settings. A previous article describing a conceptual framework for dysphagia instruction similarly describes improved clinical dysphagia content understanding with increased dysphagia management experiences [14]. This would explain why students with previous dysphagia patient exposure also scored high on DCVT self-competency ratings related to General Skills and Direct Patient Care domains. Despite the impact of previous exposure to theoretical dysphagia content during undergraduate studies appearing to impact self-competency scores, the student’s year of study did not appear to impact the self-perceived competency rating highlighting the need for dysphagia-specific patient interactions for learners.

Notably, a significant difference in DCVT self-competency ratings was noted in general skills but not in direct patient care with survey timepoint. The study conducted by Hazelwood and colleagues demonstrated a difference in direct patient care self-competency ratings with survey timepoint, but this was likely due to a difference in the timeline of administering the survey compared to the present study [2]. Students enrolled in the master's speech-language therapy program in their study completed the DCVT before each semester, spanning the course of their entire graduate school training.

The students in the master’s program at Addis Ababa were administered the DCVT before and after a one-month dysphagia-focused program. The course taught in the present study took place over only one month, contributing to general knowledge that requires more time and patient exposure to build upon to feel comfortable with providing direct patient care. Additionally, there was a language barrier between clinical instructors and non-English speaking patients. As a result, SLT students interpreted for the instructors and patients during their clinical training sessions, possibly presenting a barrier to efficient learning. Although clinical experiences were incorporated into the one-month dysphagia course, there was a limited variety of conditions that the students were able to see. Across many semesters of speech-language therapy graduate training, students are not only exposed to multiple dysphagia patients requiring similar management but also mature generally as SLT providers. As students mature as providers, they can better utilize their own patient experiences to improve their practice, contributing to confidence in their ability to care for patients with dysphagia.

The combination of both first and second year students may have influenced the results of the DCVT-DPC. Due to a lack of instructors and limited didactic time, first and second year students were combined for the one month intensive dysphagia course. Second year students have more exposure to acquired language and communication disorders (i.e. dysphagia following a stroke) than first year students as they progress through the SLT curriculum. Because of this, second year students likely had more foundational SLT knowledge and prior dysphagia patient exposure, both factors that were shown to significantly affect the results of the DCVT. Although resources preclude the course from being split into two sections for first and second year patients, respectively, the course may be restructured to overcome significant knowledge gaps between first and second year students. For example, clinical history taking and oral motor examinations are skills that can be introduced earlier in the course alongside theoretical instruction on medical diagnoses that present with dysphagia. This may allow students to become more comfortable with the components of the dysphagia evaluation and focus on the development of treatment plans during in-hospital clinical instruction. This structure would more closely mirror dysphagia course structure in SLT programs in North America, where students typically attend a one semester dysphagia didactic course followed by university clinic experience and clinical internships.

Due to the limited access to instrumental assessment of dysphagia in Ethiopia, only two of the DCVT domains were included in the present study. Specifically, skills related to videofluoroscopic swallow studies (VFSS), fiberoptic endoscopic evaluation of swallowing (FEES), and high-resolution manometry (HRM) were not assessed in this cohort of students. VFSS is not performed at Black Lion Hospital due to the high cost of radiographic equipment and difficulty accessing barium and thickening agents used in these studies. Additionally, SLTs at Black Lion Hospital do not routinely perform flexible fiberoptic endoscopic or laryngoscopic exams, with otolaryngologists and some oncologists performing the majority of scope exams. To this end, the focus of the current dysphagia curriculum was to train students to be proficient bedside generalists as opposed to focusing on instrumental evaluation methods that are not currently accessible at Black Lion Hospital. There is currently an effort to introduce the use of instrumental assessment tools during SLT care at the hospital. To help students become familiarized with these tools, future dysphagia instruction can utilize learning platforms/simulation tools such as SimuCase or online interactive VFSS or FEES learning [15, 16].

The ultimate goal of this dysphagia course and the SLT master’s program at Addis Ababa University is to train a group of practicing SLTs to not only provide patient care but also help train future SLTs in the region. Currently, there are no practicing SLTs at Black Lion Hospital that focus on dysphagia care. There is an opportunity for an SLT to join the neurology team at the hospital to evaluate dysphagia in patients with neurologic diseases, and this person may serve as a partner of this program in the future to reduce dependence on external clinicians. Establishing an effective framework for the development of dysphagia education, including the use of validated self-assessment tools, varied patient experiences, and access to online simulation learning, may allow for further development of SLT dysphagia-related practice in Addis Ababa.

There are certain limitations that should be considered with the results of this study. First, we enrolled all students admitted into the program, however given the small sample size, we cautiously interpret the findings from the 19 individuals who participated. Second, there was significant variation within the cohort of students included in this dysphagia course, as some students were already practicing health care providers while others were not. Even within the group of students who previously worked in healthcare, there was significant variation in the positions they held, contributing to differing baselines of clinical knowledge. This can be considered similar to differences in pre-clinical volunteer opportunities amongst students in speech language therapy master’s level programs in North America; however, a difference in career-related experience can be more difficult to quantify than differences in volunteer hours. In addition, patients at Black Lion Hospital were speaking in Amharic and other local languages, while the supervising SLTs communicated in English. As a result, SLT students provided interpretation to both patients and instructors, with students interpreting what patients said to the instructors, instructors providing assessment questions to students in English, and students interpreting these questions to evaluate patients. This might limit clinical learning, potentially also contributing to a lack of significant difference being seen in direct patient care with the survey timepoint. As more locally trained clinicians begin to practice and contribute to dysphagia education, this may no longer present as a limitation to clinical learning in the future.

## Conclusion

This study explored the education of master’s level trained Speech Language Therapy students in Ethiopia. The current public healthcare system in Ethiopia does not routinely integrate dysphagia trained SLTs into their teams; therefore, the medical education of these practitioners is pivotal. The DCVT is a useful tool in furthering the establishment and development of dysphagia education programs in this environment, not only allowing instructors to identify gaps in dysphagia-related training but also helping students identify areas of strength and focus on areas for improvement during their practicum. This study highlights student education in low- and middle-income countries (LMICs) and their experiences as they were provided direct patient-facing experiences during their training. This approach to training showed that regardless of DCVT domain, SLT graduate student clinicians were more likely to perceive themselves as “adequate” in their ratings at the second time point following clinical interactions. SLT Training programs, and particularly those in LMICs, are encouraged to provide increased exposure to patients with dysphagia to support increased self-perceived competency scores.

## Supplementary Information

Supplementary Material 1.

## Data Availability

The data presented in this study are not publicly available to protect the participants’ privacy, data are available from the corresponding author upon reasonable request.

## Abbreviations

SLT: Speech Language Therapy
DCVT: Dysphagia Competency Verification Tool
DCVT-GS: Dysphagia Competency Verification Tool—General Skills
DCVT-DPC: Dysphagia Competency Verification Tool Direct Patient Care
LMICs: Low- and middle-income countries
AAU: Addis Ababa University
