# Supplementary material for: Dysphagia education in Addis Ababa, Ethiopia: student self-competency ratings during their dysphagia course
Source: BMC Med Educ. 2025 May 21;25:737. doi: 10.1186/s12909-025-07365-7 (PMC12093859; doi:10.1186/s12909-025-07365-7)
Supplement: Supplementary file 1 — Supplementary Material 1. [file 12909_2025_7365_MOESM1_ESM.docx]

| **Dysphagia Student Questionnaire** | | | | | | | |
| --- | --- | --- | --- | --- | --- | --- | --- |
| **Demographic Information** | | | | | | | |
| Q1 | Student Name |  | | | | | |
| Q2 | Current Date |  | | | | | |
| Q3 | How long have you been an SLT/SLT student (if you have an undergrad in SLT please include these years)? |  | | | | | |
| Q4 | Which batch are you currently in? |  | | | | | |
| Q5 | Indicate your age |  | | | | | |
| Q6 | Indicate your sex |  | | | | | |
| Q7 | As an estimate, how many patients with dysphagia have you provided assessment, management or treatment for so far? |  | | | | | |
| **Please rate the following aspects of your dysphagia knowledge.** | | | | | | | |
| **General Skills** | | **Strongly Disagree** | **Disagree** | | **Neutral** | **Agree** | **Strongly Agree** |
| Q1 | Describes relevant research on normal swallowing |  | |  |  |  |  |
| Q2 | Explains strengths and limitations of clinical examination, including ability to detect aspiration and determine treatment strategies for pharyngeal swallowing disorders |  | |  |  |  |  |
| Q3 | Describes the etiology contributing to feeding and/or swallowing disorders |  |  | |  |  |  |
| Q4 | Identifies cognitive, communication, behavioral, and psychological factors contributing to feeding and/or swallowing status |  |  | |  |  |  |
| Q5 | Describes the potential effects of common medications on swallowing |  |  | |  |  |  |
| Q6 | Describes the interrelationships of the oral, pharyngeal, and esophageal phases of swallowing |  |  | |  |  |  |
| Q7 | Describes cross-system relationships that influence feeding and/or swallowing (e.g., respiratory, gastrointestinal, neurological) |  |  | |  |  |  |
| Q8 | Identifies signs and symptoms of feeding and/or swallowing disorders |  |  | |  |  |  |
| Q9 | Describes nutritional intake methods (oral and non-oral) and the problems associated with each that may contribute to dysphagia or be exacerbated by dysphagia |  |  | |  |  |  |
| Q10 | Collaborates with relevant team members regarding patient care |  |  | |  |  |  |
| Q11 | Describes and integrates evidence-based practice into patient assessment and care |  |  | |  |  |  |
| Q12 | Recognizes medical contraindications of proceeding with direct assessment, signs of patient distress, and necessary response |  |  | |  |  |  |
| Q13 | Describes differences between screening and assessment |  |  | |  |  |  |
| Q14 | Describes indications and contraindications for instrumental swallow study referral |  |  | |  |  |  |
| **Direct Patient Care** | | **Strongly Disagree** | **Disagree** | | **Neutral** | **Agree** | **Strongly Agree** |
| Q1 | Obtains comprehensive medical and dysphagia history, including nature and duration of signs and symptoms, prior dysphagia evaluation or treatment, response to treatment, and cultural and/or linguistic factors that may influence the patient’s preferences and attitudes toward feeding and/or swallowing |  |  | |  |  |  |
| Q2 | Determines baseline and current nutritional intake (e.g., positioning, feeding dependency, environment, diet modification, compensations) |  |  | |  |  |  |
| Q3 | Identifies when swallowing assessment and intervention is appropriate |  |  | |  |  |  |
| Q4 | Conducts an oral, pharyngeal, laryngeal, cranial nerve, and respiratory function examination as it relates to functional assessment of feeding and/or swallowing |  |  | |  |  |  |
| Q5 | Identifies abnormal/atypical structure and function |  |  | |  |  |  |
| Q6 | Assembles the appropriate assessment materials (e.g., nipples, bottles, utensils, cups, foods/liquids) as per facility-specific protocol |  |  | |  |  |  |
| Q7 | Identifies significant signs, symptoms, medical conditions, and medications pertinent to dysphagia during clinical assessment |  |  | |  |  |  |
| Q8 | Recognizes clinical signs and symptoms of airway compromise |  |  | |  |  |  |
| Q9 | Tests interventions, including but not limited to postural changes, behavioral changes, maneuvers, bolus modifications (e.g., texture, volume), delivery method (e.g., spoon, cup, bottle, nipple type), and sensory enhancement techniques to improve safety and efficiency of the swallow and trials, as appropriate |  |  | |  |  |  |
| Q10 | Refers for appropriate diagnostic tests, including instrumental swallow assessment, and consultations when indicated |  |  | |  |  |  |
| Q11 | Provides recommendations regarding delivery of nutrition and hydration (oral, non-oral, or combination of the two) |  |  | |  |  |  |
| Q12 | Provides recommendations regarding specific oral intake modifications (e.g., volume, viscosity, texture, etc.) |  |  | |  |  |  |
| Q13 | Provides recommendations regarding compensatory and feeding precautions (e.g., strategies, positioning, assistance, supervision, etc.) |  |  | |  |  |  |
| Q14 | Provides recommendations regarding rehabilitation treatment targeting physiologic deficits identified on assessment, utilizing evidence-based techniques when available |  |  | |  |  |  |
| Q15 | Integrates and adapts plan of care to include patient’s cultural and personal preferences |  |  | |  |  |  |
| Q16 | Provides a prognostic statement |  |  | |  |  |  |
| Q17 | Educates the patient and family/caregiver to the findings and recommendations, including options and relative risks/benefits |  |  | |  |  |  |
| Q18 | Educates the staff (e.g., physicians, nurses/CNAs, care planning team, teachers, aides) as to findings and recommendations, and advocates for swallowing-related services |  |  | |  |  |  |
| Q19 | Generates documentation that is clear, concise, complete, and interpretive (e.g., assessment performed/findings, impression, severity, prognosis, recommendations, and goals) |  |  | |  |  |  |
| Q20 | Identifies necessary follow-up care, including frequency of treatment, monitoring, and/or reevaluation |  |  | |  |  |  |
| Q21 | Provides ongoing assessment and revises treatment goals as appropriate, based on patient response |  |  | |  |  |  |
| Q22 | Develops and implements treatment plan targeting physiologic deficits identified on assessment |  |  | |  |  |  |
| Q23 | Documents response to treatment using objective and measurable data collection systems |  |  | |  |  |  |
| Q24 | Adjusts treatment plan, content and delivery to the level of the person being educated, counseled, or trained |  |  | |  |  |  |
| Q25 | Identifies discharge/dismissal criteria |  |  | |  |  |  |
| Q26 | Seeks assistance and collaboration as needed in the assessment and care of persons with dysphagia |  |  | |  |  |  |
| Q27 | Describes best practices for providing interventions when complicated and/or special medical conditions are seen which may have an impact on an individual’s feeding and swallowing |  |  | |  |  |  |
